# Cystic angiomatosis, pleural effusion and multiple bone lesions mimicking a metastatic malignant neoplasia: a case report

**DOI:** 10.1186/s13256-019-2196-3

**Published:** 2019-08-22

**Authors:** Caroline Souza dos Anjos, Rafaela Melo Campos Borges, Ananda Castro Chaves, William Hiromi Fuzita, Carlos Augusto Moreira Silva, Ubiratã Gomes Loureiro de Oliveira

**Affiliations:** 1 Oncologic Therapy and Imaging Diagnosis Clinic - SENSUMED-Oncologia, Rua Prof. Marciano Armond, 545 - Adrianópolis, Manaus, AM Brazil; 2 Internal Medicine, Hospital Adventista de Manaus, Av. Gov. Danilo de Matos Areosa, 139 - Distrito Industrial, Manaus, AM Brazil; 3 Institute of Surgical and Molecular Pathology - IPCM Belém, Tv 14 de março 1155, sala 1304 Umarizal, Belém, PA Brazil

**Keywords:** Angiomatosis, Pleural effusion, Osteolytic lesion

## Abstract

**Background:**

We report a case of a patient with a rare clinical condition: cystic angiomatosis presenting as pleural effusion and multiple bone lesions mimicking a metastatic malignant neoplasia. With only about 50 such cases published in the literature, it is important to report the clinical presentation and proposed treatment and to share information about the clinical evolution in these patients.

**Case presentation:**

We report a case of a 45-year-old white man who presented to our hospital with ventilator-dependent pain. Chest tomography detected pleural effusion and multiple osteolytic bone lesions. Oncologic investigation for metastatic malignant neoplasia was started after exclusion of an infectious process. Imaging examinations revealed diffuse osteolytic lesions as well as cystic lesions of the spleen, with discrete glycolytic hypermetabolism visualized by positron emission tomography. After negative results were obtained by investigation of the primary tumor site and a bone biopsy, a final diagnosis of cystic angiomatosis was made.

**Conclusions:**

In view of the fact that cystic angiomatosis is a heterogeneous disorder of unpredictable prognosis and uncertain treatment, it is necessary to disseminate new cases so that further studies may be undertaken to obtain further physiopathological findings and an effective treatment.

## Introduction

First described by Jacobs and Kimmelstiel in 1964, cystic angiomatosis is an extremely rare condition characterized by multifocal dissemination of hemangiomatous or lymphangiomatous skeletal lesions affecting the axial and appendicular skeleton, with the possible involvement of visceral organs [1, 2]. The pathogenesis of angiomatosis continues to be obscure and uncertain, with the condition being considered to result from vascular malformations of congenital origin [1, 3].

The disease is frequently asymptomatic and is incidentally detected on radiographs obtained for different reasons [1, 4]. The imaging characteristics of systemic cystic angiomatosis may mimic skeletal involvement of secondary malignant neoplasia, with the condition thus being erroneously diagnosed as such [1]. Therefore, it is essential to carefully consider pathological changes in cases of disseminated angiomatosis in view of the fact that the radiological appearance may resemble that of metastatic malignant tumors.

The skeletal lesions of generalized cystic angiomatosis are usually stable or may regress spontaneously. Visceral lesions, although considered to be benign, may cause fatal complications during the natural history of the disease [1]. Thus, during the diagnostic investigation, it is essential to perform differential diagnosis for malignant neoplasia.

In view of the rarity of this clinical condition, with about 50 cases reported in the literature, we report a new case of cystic angiomatosis with visceral involvement in a patient with an initial clinical presentation as plural effusion. The clinical presentation, diagnostic stages, and treatment of this patient are described.

## Case presentation

A 45-year-old white man with no previous comorbidities developed flulike signs and symptoms (rhinorrhea, dry cough, holocranial headache, and myalgia) of about 5 days’ duration. He was, a nondrinker and nonsmoker. After resolution of his symptoms, dry cough and right pleuritic pain persisted. Then, he sought medical care with these complaints, and his physical examination plus pulmonary auscultation revealed abolished vesicular murmur on the right base. Laboratory examinations such as blood count, electrolytes, and hepatic and renal function showed no changes, whereas a posteroanterior and profile chest radiograph revealed plural effusion on the right side. On that occasion, he denied dyspnea, fever, weight loss, or other associated symptoms. Regarding his family history of morbidity, he reported the death of a 49-year-old brother due to complications of a metastatic renal neoplasia.

**Fig. 1:**
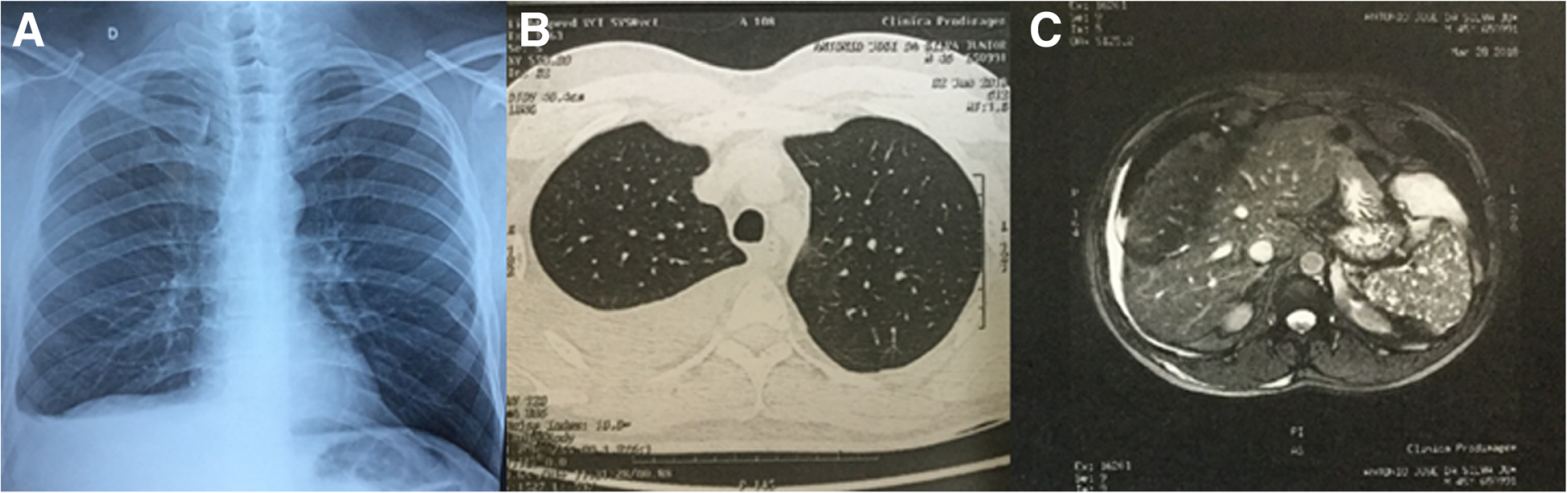
**a** Posteroanterior (PA) chest x-ray showing pleural effusion on the right. **b** Chest computed tomography with contrast enhancement showing pleural effusion in the right hemithorax. **c** Magnetic resonance imaging (MRI) showing multiple lytic lesions in the spleen parenchyma

After an initial clinical evaluation at our internal medicine outpatient clinic for the differential diagnosis of the etiology of pleural effusion, he underwent diagnostic thoracocentesis (Table 1) and chest computed tomography for diagnostic elucidation. Chest computed tomography revealed pleural effusion associated with circumscribed osteolytic lesions distributed along the bone scaffold, more clearly visible in the sternum and in a vertebral segment (Fig. 1). Next, the patient was referred for clinical oncology evaluation in view of the clinical suspicion of a malignant neoplasia with bone metastasis.

**Table 1:** Analysis of pleural fluid

Test	Value	Test	Value
pH	8.5	Total proteins	5.3 g/dl
Density	1.015	Lactate dehydrogenase (LDH)	221 IU/L
Color/aspect	Yellow/turbid	Pleural fluid protein/protein serum	0,74
Nucleated cells	10/mm^3^	Pleural LDH/serum LDH	1,09
Red blood cells	60/mm^3^	Glucose	125 mg/dl
Leukocytes - lymphocytes	100%	Bacterioscopy	Leukocytes and rare epithelial cells
Mesothelial cells, histiocytes, neutrophils, eosinophils	Absent	BK and Xpert MTB/RIF tests	Negative
Amylase	55 IU/L	Oncotic cytology	Negative for atypical cells

*BK* BK virus, *Gene Xpert MTB/RIF* Rapid molecular method based on the polymerase chain reaction for the detection of *Mycobacterium tuberculosis* with resistance to rifampin

**Fig. 2:**
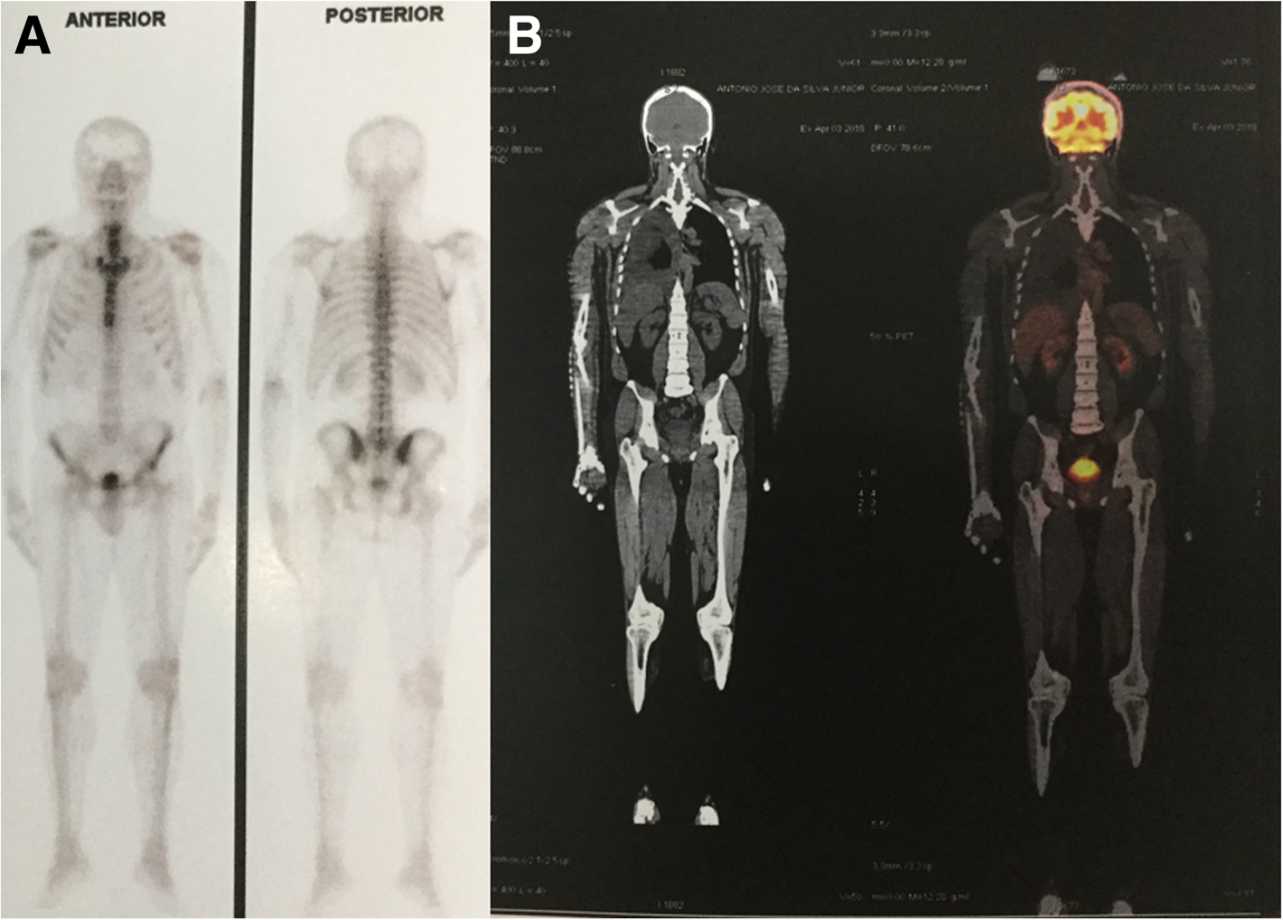
**a** Bone scintigraphy with heterogeneous distribution of the radiopharmaceutical M-methylenediphosphonate technetium-99m throughout the skeleton, with hyperconcentration of the tracer in the shoulders suggesting degenerative changes. **b** Positron emission tomography-computed tomography with fluorodeoxyglucose demonstrating multiple lytic bone lesions without a sclerosis halo or cortical bone lysis, some of them showing discrete glycolytic hypermetabolism (maximum standardized uptake value, 2.9)

In view of the evidence of bone lesions, complementary examinations were requested in order to screen for a possible metastatic malignant neoplasia. The patient’s prostate-specific antigen value was within normal limits; protein immunofixation showed the absence of a monoclonal band; and results of serology for hepatitis B and C and human immunodeficiency virus were negative. Additionally, the patient’s alkaline phosphatase, parathyroid hormone, and vitamin D were normal. Bone scintigraphy showed hyperconcentration of the tracer in the shoulders, suggesting degenerative changes, and positron emission tomography-computed tomography with fluorodeoxyglucose demonstrated multiple lytic bone lesions without sclerosis halo or cortical bone lysis (Fig. 2).

**Fig. 3:**
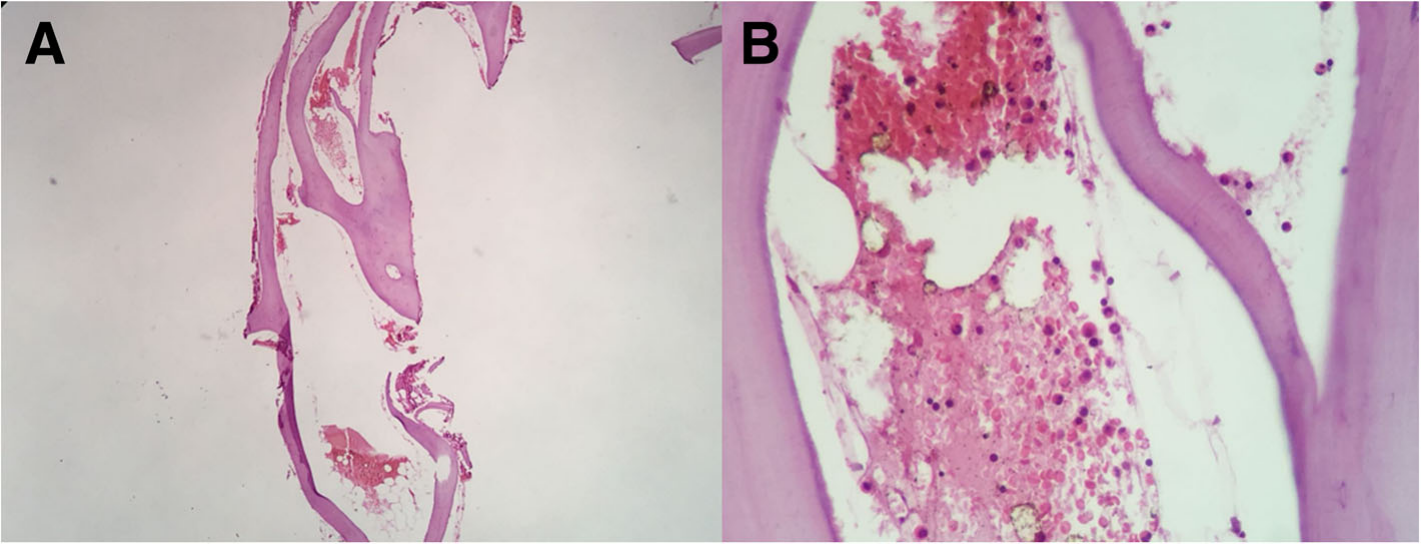
Histopathological examination of a bone biopsy. **a** Panoramic view of the bone biopsy showing lacunar areas with hemorrhagic content and an apparent thin membrane. **b** Fragment with intense myeloid hypocellularity and presence of lacunar areas with a hemorrhagic content

In view of the lack of evidence of neoplastic disease after the complementary examinations, a bone biopsy was indicated in order to complete the diagnostic investigation. A bone biopsy of the left acetabulum revealed the presence of a bone fragment with preserved and mature cellularity. A biopsy of the left proximal femur showed intense myeloid hypocellularity and lacunar areas with hemorrhagic content, although without the characterization of a cystic area with an endothelial lining (Fig. 3). Both biopsies revealed the absence of myeloid neoplasia, lymphoma, or carcinoma in the sample.

A diagnosis of cystic angiomatosis was made on the basis of imaging and bone biopsy criteria correlated with the presence of osteolytic lesions and a negative investigation of neoplasia. The patient continued to be asymptomatic 2 months after the diagnosis, with permanence of pleural effusion and without progression of lytic lesions or laboratory changes. Treatment with zoledronic acid 4 mg intravenously every 28 days was started in order to stabilize the bone lesions and to prevent skeletal events such as pain and fracture. 

## Discussion and conclusions

Cystic angiomatosis is an extremely rare condition characterized by multifocal dissemination of hemangiomatous or lymphangiomatous skeletal lesions affecting the axial and appendicular skeleton, with the possible involvement of visceral organs [1, 2]. The first manifestations of cystic angiomatosis occur during the first decades of life, particularly during puberty. However, some authors have reported cases of late onset diagnosed after 60 years of age, suggesting that there may be a second peak of occurrence. The literature includes a reported case of a patient diagnosed at 52 years of age, although the median age at diagnosis of the 48 cases described to date is 22.8 years [3, 5, 6]. The initial clinical presentation of our patient involved pleural effusion and asymptomatic bone lesions, a presentation that was not observed in the previous literature reports.

The thoracic spine is the most common site of vertebral involvement. Although skeletal angiomatosis may occur as a single condition, concomitant disease of nonosseous tissues mainly involve the spleen, as reported in a literature review in which 12 of the 48 cases described had cystic spleen lesions, as also observed in our patient [1, 3]. Other visceral sites possibly involved are lung, liver, and spleen, as observed in up to 60–70% of the cases reported [1, 2]. Men are more frequently affected than women, with an incidence ratio of approximately 2:1 [3].

Images commonly reveal lesions with typical characteristics such as multifocal intramedullary skeletal cysts with relatively well-preserved cortical bone and no periosteal reaction. The cysts are oriented along the long axis of the bone and are classically surrounded with a sclerotic peripheral ring. Histological examination typically reveals vascular canals with a single layer of flattened endothelial cells [3].

No peripheral involvement of soft tissues or periosteal reaction is observed, facilitating the differential diagnosis of metastasis, multiple myeloma, or other malignant conditions. Cystic angiomatosis shares features with Gorham-Stout disease but differs from it in relevant aspects. Angiomatosis has a better prognosis. The margin of the cysts has a sclerotic appearance in radiographs and sclerosing lesions rather than osteolysis, which may not be observed [3].

The diagnosis of systemic cystic angiomatosis is made using a bone biopsy. However, literature reports have demonstrated that histological diagnosis is difficult, and very frequently many biopsies are needed to reach a final diagnosis [2]. Histology reveals vascular canals, vascular cysts, vascular spaces and cavities, hemangiomas, and lymphangiomas [2, 4]. The biopsy of our patient showed lacunar areas with hemorrhagic content, although it had no cystic wall of endothelial lining, which has been reported in all other cases in the literature. Due to difficult access from the site of the bone lesion for biopsy and before the radiological signs, it was decided not to repeat the procedure. Because of the low specificity of pleural biopsy, the medical team opted not to carry out the procedure.

The treatment of cystic angiomatosis is support and is directed at the control of symptoms, involving fracture fixation or excision of soft tissue lesions. The use of bisphosphonates, radiation, and interferon, as well as clinicoradiological patient follow-up for visualization of the spontaneous interruption of lesions, has also been reported [6]. Bisphosphonates reduce osteolysis and increase bone mineralization. Zoledronic acid controls the expression of angiogenic cytokines, modulating the migration and adhesion of endothelial cells. This probably stabilizes the bone lesions and causes fewer skeletal events and was the option of preventive treatment for our patient because he was asymptomatic. Radiotherapy and surgical treatment of complications are also used for the treatment of this disease. However, the real benefit of any type of treatment is not clear [7].

In our patient, cystic angiomatosis with visceral involvement exhibited pleural effusion, which, to the best of our knowledge, is the first such case reported in the medical literature. The rarity of the disease and its clinical presentation demonstrate the importance of reporting experiences with the condition, because increased clinical suspicion will prevent unfavorable patient outcomes.

Cystic angiomatosis, a rare disease involving the vascular and lymphatic systems, should be included in the differential diagnosis in the investigation of a metastatic malignant neoplasia with no evidence of the primary site. In view of the fact that this is a heterogeneous disorder of unpredictable prognosis and uncertain treatment, it is necessary to disseminate new cases so that further studies may be undertaken in order to obtain further physiopathological findings and an effective treatment.

## Data Availability

Clinical data and complementary examinations are available according to authorization by the patient.

## Abbreviations

Gene Xpert MTB/RIF: Rapid molecular method based on the polymerase chain reaction for the detection of *Mycobacterium tuberculosis* with resistance to rifampin
